# News media coverage of the U.S. social safety net: themes and gaps from a scoping review

**DOI:** 10.1186/s12889-025-23875-x

**Published:** 2025-09-30

**Authors:** Sarah E. Gollust, Quin Mudry Nelson, Carson Crane, Yusra Murad, Margaret E. Tait

**Affiliations:** 1 Division of Health Policy and Management, University of Minnesota School of Public Health, 420 Delaware Street SE, MMC 729, Minneapolis, MN 55455 USA; 2 Department of Health Studies, University of Richmond, Richmond, VA USA

**Keywords:** News media, Social safety net, Poverty, Health insurance, Housing, Welfare, Paid family and medical leave, Early childhood education

## Abstract

**Background:**

The social safety net, the set of policies and programs designed to improve the quality of life of individuals facing periodic or persistent financial hardship, is critical to improving population health. Despite strong evidence of its importance, policy investments in the social safety net in the United States continue to face political obstacles. One important contributor to the political dynamics of issues is public opinion, which can be shaped – for better or worse – by the news media. By reporting on, interpreting, and contextualizing policy issues, the news media play a key role in shaping public understanding of social issues.

**Eligibility criteria:**

In this scoping review, we summarize cross-disciplinary literature that examined news media coverage of social safety net issues (*N* = 40 studies).

**Results:**

We find that news coverage often focuses on individual stories and infrequently connects to broader, systemic issues, missing the opportunity to garner public support for the social safety net. Across studies, authors seldom note the implications of their work for journalistic practice nor for health equity.

**Conclusions:**

Future work should examine how population health research evidence can be shared with journalists and news producers for more comprehensive media attention to the safety net within the context of the constraints news producers face.

**Supplementary Information:**

The online version contains supplementary material available at 10.1186/s12889-025-23875-x.

## Background

Decades of research confirm the importance of the social determinants of health – including resources like income, housing, and early education – in shaping the distribution of population health in the United States [1]. Accordingly, research also demonstrates that investments in the social safety net are promising interventions to improve population health and advance health equity [2]. By the social safety net, we are referring to the set of policies that support and improve the lives of people with low incomes or those experiencing short-term economic hardship [3]. More specifically, for the purposes of this study, the social safety net includes policies and programs to support individuals’ and families’ income (e.g., welfare programs), health insurance (e.g., Medicaid and/or access to health insurance Marketplaces), food access (e.g., Supplemental Nutrition Assistance Program or SNAP), housing affordability, paid family and medical leave, and early childhood education.

Research in population health has long suggested that the social safety net is a critical set of supports that can improve health and well-being in individuals and communities (e.g., [2]). In a landmark report in November 2023, these links between social conditions and associated social policies were recognized at the federal level by the Biden Administration’s publication of the “Playbook to Address Social Determinants of Health” [4]. In spite of this recognition, demonstrable federal policy action to make the social safety net more robust in the United States has been politically-contested at Best, and actively opposed at worst. For instance, the Bipartisan Infrastructure Law signed into law in November 2021 provided an infusion of funds for roads, bridges, rail transit, and broadband, but left out a proposal to expand Medicaid access to long-term care and home and community-based services for disabled people [5]. Similarly, the Build Back Better Act, which would have incorporated new social safety net investments such as universal pre-kindergarten and paid family leave, stalled out in Congress. When elements of the bill ultimately ended up in the Inflation Reduction Act of 2022, neither the early childhood nor paid family leave elements were included. Further, while the public tends to support Medicaid (the public health insurance program for those in poverty) [6], Republican legislators in a handful of mostly southern states continue to resist expanding access. In 2025, Medicaid faces Renewed funding threats. The One Big Beautiful Bill Act, the budget reconciliation bill of 2025, includes $806 billion in proposed cuts to Medicaid over ten years. These cuts would not only reduce the number of people who rely on Medicaid, but also impose logistical and financial barriers for those who remain covered, including work requirements to maintain coverage and increased cost-sharing for people with incomes above the federal poverty line [7].

One important factor that contributes to the political dynamics of policy investments in the social safety net is public opinion, how the public views the policy problems, solutions, and the target populations affected by those problems. The ways the news media frame these issues can be an important influence on public perceptions and thus offer insight into the political dynamics of efforts to promote further investment into the safety net. However, empirical research on news media coverage of social issues tends to focus only on single issues, and the research is published in diverse disciplinary channels. Such fragmented dissemination of evidence limits our ability to draw conclusions about aggregate themes of news media coverage across multiple social safety net topics. When published research has diverse study designs across many topical areas, scoping reviews can be helpful to organize the evidence and characterize the extent and range of existing research [8]. Scoping reviews are particularly useful when other more structured approaches, like systematic reviews, are not appropriate given the diversity of available evidence. The current study provides an overview of U.S. news media coverage of a set of key social safety net policies, to draw conclusions about the contemporary politics of the social safety net in the United States.

### News media influence on policy

The news media can influence the public’s perceptions of policy matters through two mechanisms: agenda-setting and framing. Agenda-setting refers to the process that determines which of many societal issues are those the public considers important at a given point in time. When the media devote more attention to certain issues over others, the public thinks those issues are more important [9, 10]. For instance, research demonstrates that the news media devote more attention to breast cancer, relative to other types of cancer [11] and to diseases with a higher disease burden among white people, compared to Black people [12], indicating that the volume of attention to news issues does not necessarily correlate with the objective measures of burden.

Framing refers to the “interpretive packages” that journalists use in presenting social issues to the public [13]. Such choices can include how to cover the definitions of social issues, the causes of those issues, moral judgements, and how to address them—particularly whether the solutions lie internally with individuals and families or externally such as in policymaking [14]. One important framing convention in news media of health and social policy issues is whether the story is framed to focus on particular individual case studies (e.g., episodic framing) or to broader historical or structural trends (e.g., thematic framing) [15, 16]. Stories that focus mainly on individuals tend to invite individualistic attributions of responsibility to address them and lower support for policy solutions [16]. Similarly, attribution theory indicates that stories that emphasize individual causes will lead to more individual-level assignment of blame and less understanding of the broader systemic factors [17].

Another important contribution of the news media to public perceptions of social policy issues is through the news media’s framing of social groups affected. Whether within news story text that describes which groups could benefit or be harmed, or in the images that accompany stories in print or on TV, the media contribute to the mental images that people carry about those affected by social issues [18, 19]. The U.S. public’s perceptions of social policy issues is “group-centric”, that is, support for a given policy often hinges on the public’s attitudes about the group affected [20]. Such attitudes often dovetail with racist stereotypes about groups, such as whether groups needing income supports are lazy or hardworking [21]. These attitudes may have particularly important influences on opinion about social safety net policies that are targeted, by design, to the most vulnerable and those with lower resources. Building off these foundational theories are more contemporary theories of stigma communication, which describe how the media can contribute to stigmatizing beliefs among the public, through messages that distinguish and categorize people (such as people receiving welfare benefits), imply that these people are responsible for their status, and link the group to a concept of peril [22]. In turn, empirical research suggests that these stigmatized identities can rapidly spread and be shared through news, and especially through social media, where short messages (such as Tweets or posts) can apply stigmatizing labels to groups rapidly, such as in the recent cases of Mpox and COVID-19 (see, e.g., [23, 24]).

### Importance of the social safety net for population health

This scoping review concerns six domains of the social safety net: poverty, welfare, and social security disability insurance (SSDI); health insurance; food assistance, hunger, and nutrition; housing and homelessness; paid leave; and early childhood care and education (ECE). While a complete review of the health consequences of each of these domains is beyond the scope of this article, evidence exists that supports the contention that policy investment in each of these domains could advance overall population health and reduce health inequity [2]. For example, research demonstrates that investments in Medicaid at early ages have contributed to long-term health benefits [25], and access to food assistance also has long-run health benefits [26]. Yet such data alone are insufficient to drive policy change. Research suggests that public opinion about social safety net policies varies by the type of policy, and has declined since the height of the pandemic [27]. Given what is known about the effects of news media coverage on the public’s perceptions, the political feasibility of expanding the social safety net is likely related to whether and how the news media present these areas as policy problems worthy of investment.

### Study objectives

When previous studies have characterized news media coverage of the social policy topics that comprise the safety net, the studies are often point-in-time assessments of a particular event (e.g., the passage of the Affordable Care Act (the ACA); a change to SNAP benefits) rather than assessments over time, and they usually examine a single policy issue. Similarly, researchers working within a particular policy space (e.g., education researchers, public health researchers) usually focus only on single domains, but the social safety net spans multiple policy arenas. A more complete understanding of the political dynamics of the U.S. social safety net thus requires assessment of media coverage across multiple issues and over time. This gap is best suited for a scoping review. Specifically, the goal of this review is to synthesize findings from past research and respond to the following questions:

1. How has the U.S. news media communicated about a set of policies that comprise the social safety net?
2. To what extent has the news media presented the equity implications of these social policy areas, or the concept that certain populations have and continue to face systemic and structural issues that prevent them from opportunities (e.g., improved health or better housing)?

## Methods

We relied on frameworks for conducting scoping reviews to guide this study [8, 28, 29]. A scoping review aims to “…summariz[e] a range of evidence in order to convey the breadth and depth of a field,” [8]. Our review did not focus on assessing the *quality* of the evidence presented; nor were included articles limited to a specific study design, key distinctions between scoping and systematic reviews [28]. While this was not a systematic review, we followed as much as possible the PRISMA guidelines in reporting [30]. This study is registered with the Open Sciences Framework (https://osf.io/uce8s/).

### Keyword searches

Keyword searches were implemented at three time points to retrieve relevant English language articles from Google Scholar and Scopus databases: in the summer of 2020, and then refreshed in the summer of 2021 and fall of 2022 to identify newly published work. Scopus includes journals, conference proceedings, and books curated by editors. Google Scholar identifies a broader selection of literature, including non-peer-reviewed reports [31]. We developed a set of keywords using the substantive area of focus (e.g., “poverty” or “child care”) with the Boolean operator “AND” followed by either “media”, “media coverage”, or “news coverage” (Appendix Table 1). These searches were implemented within article titles, abstracts, and keywords.

### Abstract review and inclusion criteria

One author (MT) completed an initial screen of abstracts to assess their inclusion based on whether they had a substantive focus on media and social safety net policy. The author reviewed the first 10 pages of results (*n* = 100) for searches that yielded more than 500 results. If a relevant article appeared on the last Pages, they reviewed an additional 5 Pages of content for a total of 150 abstracts for each keyword search. These searches led to a total of 3,980 potentially relevant abstracts. Abstracts that explored social safety net policy but were not related to media were excluded at this point (*n* = 3,897 exclusions); the vast majority of articles identified only had a passing reference to media rather than a focus on news content.

**Fig. 1:**
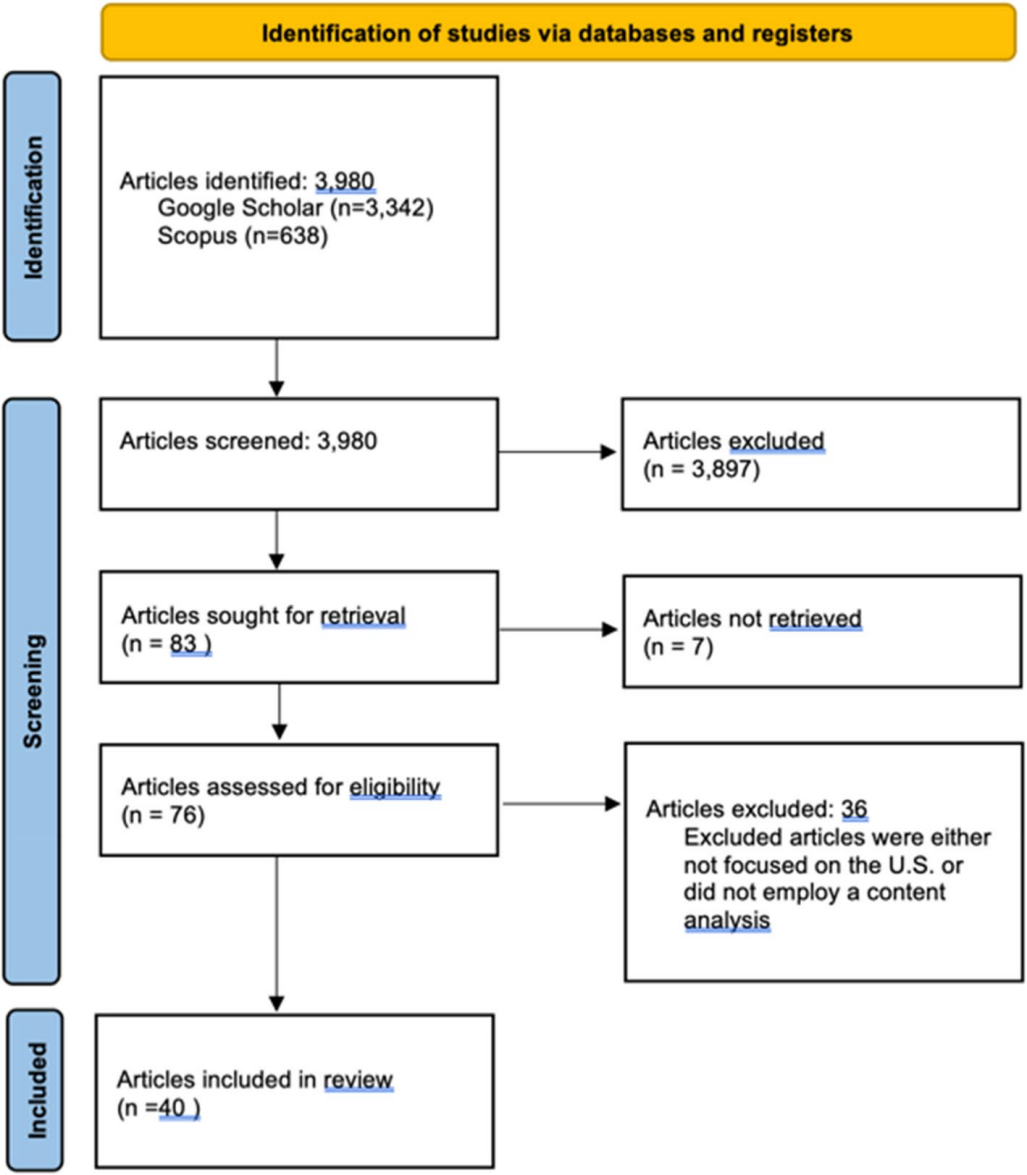
Flow diagram for scoping review. Figure note: Flow diagram based on PRISMA guidelines; [30]

Full text articles for the remaining 83 abstracts were retrieved and reviewed by a team of 4 trained research assistants. Additional exclusions were applied at this stage (see Fig. 1): strictly conceptual or analytic essays (e.g., without a presentation of empirical data); empirical work describing news media exclusively outside of the U.S.; and media coverage of a particular disease or condition, without a focus on policy solutions. Critical or rhetorical analyses and case studies that did not use a systematic approach to sampling and data collection were also excluded. Studies with evidence drawn not from news media itself but from interviews with journalists were also excluded. Finally, theses, dissertations, and conference proceedings not published elsewhere or research reports without accessible full text were excluded.

### Data collection

We created and implemented an instrument (Appendix Table 2) that trained team members used to collect data from each of the included articles, with the following fields of data collected: year of publication; number and discipline of authors; study design (i.e., qualitative, quantitative, or mixed methods); type of content analysis (i.e. human coding or machine-based); time period of included data; type of media analyzed; geography represented; main topic; whether or not the article focused on COVID-19; and the research questions assessed. During this full-text review (*n* = 83), we excluded additional articles (as described in criteria above); 43 articles were excluded, and the final sample included 40 articles.

The instrument also included prompts to describe the key findings and implications for policy or practice. Research assistants independently completed these qualitative fields based on the content of the article. A separate field (“Based on your perspective as an analyst, what are the implications of this research?”) surfaced team members’ own interpretive insights.

### Analysis

Simple descriptive statistics (frequencies) were calculated for all categorical data fields collected. For the qualitative fields, two study authors were assigned to each safety net topic and reviewed the qualitative output from the instrument. Each pair wrote up an analytic memo summarizing the key themes within a particular topic; these memos were then further synthesized as qualitative findings presented below.

## Results

### Description of studies on news media coverage of the U.S. social safety net

Articles (*N* = 40) were published between 1990 and 2022 and authored by between one and six authors (median author team was two authors). Disciplines represented by the authors (e.g., institutional affiliations) were broad, and included advocacy, anthropology, Asian American studies, communication/journalism/media studies/advertising, computer science, genetics and cell biology, nutrition, public health and health science, political science/government, public policy and public affairs, and sociology. The most common disciplinary orientation was mass communication or journalism. The articles were published in a wide range of journals, from health journals (e.g., *JAMA, American Journal of Public Health*) to political science (e.g., *Public Opinion Quarterly, Political Behavior*), to journalism/communication (e.g., *Mass Communication and Society*, *Newspaper Research Journal,*) to more-targeted topic-specific journals (e.g., *Food Studies, Journal of Aging & Social Policy*); four were non-peer-reviewed reports (Appendix Table 3).

While study publication dates ranged from 1990 to 2022, the studies reported on news coverage published from 1960–2020, with most of the studies covering 1993–2019 (Appendix Table 3). The majority (60%) relied on mixed methods, applying quantitative content analysis but with at least some qualitative interpretation (Table 1). Most (87.5%) applied human coding methods as opposed to machine coding or computational approaches. The majority (75%) examined print news media (or their online analogs, newspaper websites); only six studies examined television news and four examined magazines. No articles reported on entertainment media or social media, as expected based on the keyword searches emphasizing news. More studies (80%) reported on national news than local news (17.5%).

**Table 1:** Descriptive summary of included articles (*N* = 40)

Variable	N (%)
Study design	
Mixed	24 (60.0)
Quantitative only	11 (27.5)
Qualitative only	5 (12.5)
Type of content analysis	
Human coding	35 (87.5)
Both human and machine coding	4 (10.0)
Machine coding	1 (2.5)
Type of media	
Print or online	30 (75.0)
TV news	7 (17.5)
Magazines	5 (12.5)
Other	1 (2.5)
Social media	0
Entertainment media	0
Geography of media examined	
U.S. national	32 (80.0)
Less than national/local	14 (17.5)
Comparative: U.S. to another country	1 (2.5)
Main topic	
Poverty/welfare/SSDI	14 (35.0)
Health insurance/Medicaid	12 (30.0)
Food assistance/hunger/nutrition	4 (10.0)
Other social policy, inequality	4 (10.0)
Housing/homelessness	3 (7.5)
Paid leave/family leave	2 (5.0)
Early childhood education	1 (2.5)
Research addresses COVID-19	
No	39 (97.5)
Yes	1 (2.5)

Numbers do not add up to 100% because many variables were “check all that apply” for any given article

The most common topic represented was poverty or welfare (14 studies), followed by health insurance (12 studies). Far fewer studies focused on food assistance, other social inequality, housing, paid leave, or early childhood education.

### Thematic findings by safety net topic area

In the content that follows, we detail the years of data and type of media analyzed across articles; key themes (Table 2); any inclusion of recommendations for journalists covering these topics; and implications of the research for health equity.

**Table 2:** Thematic assessment of key findings by topic area

Topic (*N* = 40)	Time range and policy topics covered	Key qualitative themes about news media content of the topic	Author and year of included articles *Full article details in appendix 3*
Poverty/welfare (*n* = 14)	1990–2017 • Pre-welfare reform • Welfare reform • Post-welfare reform	Episodic versus contextual framing Target population framing, stereotypes, misperceptions, deservingness	• Iyengar, S. (1990) [32] • Gilens, M. (1996) [33] • Clawson, R. and Trice, R. (2000) [34] • Lawrence, R. (2000) [35] • Schram, S.F., and Soss, J. (2001) [36] • Yoo, G.J. (2002) [37] • Luther, C., Kennedy, D., & Coomb-Orme, T. (2005) [38] • Dyck, J. And Hussey, L. (2008) [39] • Kelly, M. (2010) [40] • Brown, H. (2013) [41] • Rose, M., and Baumgartner, F.M. (2013) [42] • van Doorn, B. (2015) [43] • El-Burki, I., Porpora, D., & Reynolds, R. (2016) [44] • Epp, D.A. and Jennings, J.T. (2020) [45]
Health insurance, Medicaid (*n* = 12)	1996–2022 • Clinton health reform • Affordable Care Act/Obamacare • Medicaid	Politicized coverage over substantive Politician sources dominate Use of values in framing (e.g., personal responsibility, compassion) Limited attention to health equity	• Dorfman, L., Schauffler, H., Wilkerson, J., & Feinson, J. (1996) [46] • Huebner, J., Fan, D., & Finnegan, Jr., J. (1997) [47] • Brodie, M., Altman, D., Brady, L., & Heberling, L. (2002) [48] • Hopper, J. (2015) [49] • Kim, S–H, Tanner, A., Foster, C., & Kim, S. (2015) [50] • Gollust, S., Baum, L., Niederdeppe, J., Barry, C., & Fowler, E. (2017) [51] • Kim, S., Tanner, A., Kim, S., Foster, C., Oh, S., & Chang, J. (2017) [52] • Viladrich, A. (2019) [53] • Biswas, M. & Kim, N. (2020) [54] • Gollust, S., Fowler, E., & Niederdeppe, J. (2020) [55] • Rozier, M. & Singer, P. (2021) [56] • Jahng, M. & Littau, J. (2022) [57]
Food assistance/hunger (*n* = 4)	2012–2022 • SNAP • School lunches	Framing matters (i.e., for introducing stigma, cueing systems thinking) Sources and political ideology shape frames in coverage Differing perspectives on the role of government in food assistance	• Tolley, N. & Ibrahim, J. (2012) [69] • Chrisinger, B., Kinsey, E., Pavlick, E., Callison-Burch, C. (2020) [58] • Spruance, L., McConkie, M., Patten, E., & Goates, M. (2021) [70] • Mejia, P., Mahmood, H., Perez-Sanz, S., Garcia, K., & Dorfman, L. (2022) [59]
Other social policy (*n* = 4)	2002–2017 • Children’s issues • Rising healthcare costs • Racial and ethnic health disparities	Framing matters for solutions Contextual information is not provided in mainstream media	• Kunkel, D., Smith, S., Suding, P., & Biely, E. (2002) [60] • Cabrera Rasmussen, A. (2014) [71] • Kim, B., Lowrey, W., Buzzelli, N., & Heath, W. (2021) [61] • Biswas, M. & Kim, N.Y. (2022) [62]
Housing and homelessness (*n* = 3)	2010–2021 • Root causes of homelessness • Policy responses to homelessness • Structural solutions (e.g., affordable housing)	Construction of homelessness as social versus individual problem Emphasis on philanthropic solutions versus structural Limited use of health sources	• Best, R. (2010) [63] • Nixon, L., Schaff, K., Meija, P., Marvel, D., & Dorfman, L. (2019) [64] • Borum Chattoo, C., Young, L., Conrad, D., & Coskuntuncel, A. (2021) [65]
Paid leave (*n* = 2)	2012–2019 • Maternity leave • Paid leave, broadly	Limited health equity appeals, more of a focus on gender equity	• Grandy, K. (2016) [66] • Tait, M., Bogucki, C., Baum, L., Franklin Fowler, E., Niederdeppe, J., and Gollust, S. (2021) [67]
Early childhood education (*n* = 1)	2000 & 2003 • General (no specific policy)	Superficial versus substantive coverage, neglecting full scope of benefits of ECE	• McAdams, K. & Henry, T. (2006) [68]

### Poverty/welfare/SSDI

Articles on this topic were published between 1990 and 2021 and included analysis of media coverage from the pre-welfare reform period (before the 1990s), the welfare reform efforts of the 1990s under the Clinton Administration (Temporary Aid to Needy Families, or TANF), and issues following this reform. Studies included content from local and national print or online newspapers and TV news. Studies also reviewed content from magazines [33–35, 39, 43], the only topic area that did so. One overarching finding was that much news media attention focuses on the political “game” and less on the substance of poverty policy, although this trend was less pronounced in state-oriented coverage [35]. Another key finding was that policy shaped media depictions, with articles noting that the passage of welfare policy tended to shift the narratives around target populations, with racialized media content becoming less explicit following the passage of TANF (see, e.g., [39, 44]).

Many articles build on Iyengar’s work (1990) to consider whether poverty was framed as an individual or structural issue, as well as the implications for how target populations are presented [34, 42]. For instance, in one study, authors evaluated the extent that news featured episodic coverage of particular people living in poverty, recognizing this as a way to further stereotypes that people benefitting from welfare programs are lazy and shirk responsibility [38].

Gilens’ [33] article on the news media’s role in fostering public misperceptions of individuals Living in poverty also inspired much of the scholarship. Gilens found that Black people made up 62% of the poor people pictured in news magazine stories but in reality comprised only 29% of the population living in poverty [33]. Authors have expanded on this scholarship to evaluate more recent news, as well as the intersections of race and gender and other selected ‘deserving’ populations. One analysis revealed that the overrepresentation of Black people in poverty persisted, while Asian and Hispanic individuals were underrepresented [34]. Findings from two studies indicate that women were more often depicted as welfare recipients [38] and content is highly racialized, often representing Black female welfare recipients [40]. Another study highlighted a shift in more recent news coverage toward more ethnic and racial diversity among public assistance program recipients [41]; a study of opinion pieces demonstrated similar shifts away from Black overrepresentation among those in poverty and more causal attributions to the economic structure and government, instead of individuals themselves [44]. Yoo [37] also highlighted how media narratives can shift as the result of policy change; older immigrants were depicted in news as an undeserving population prior to the passage of federal welfare reform but after this population was eligible to benefit from Supplemental Social Security Income, coverage shifted to view them as deserving [37]. Another study catalogued tone of coverage of the late-1990s TANF reform, noting that most media coverage was framed to emphasize the success of these policies [36]—possibly in response to earlier news coverage that emphasized the undeservingness of recipients prior to policy change.

No articles included any explicit reference to income supports and health, indicating the absence of health equity frames. In their conclusion, Clawson and Trice [34] emphasized the importance of journalists humanizing people depicted as in poverty. The most recent news content included was from 2015 [45], the dearth of recent evidence makes it challenging to ascertain whether depictions of people facing poverty and using welfare programs have shifted to become more sympathetic or to include more health-relevant frames.

### Health insurance/Medicaid

Articles within this topic were published from 1996 to 2022, covered specific policies or the topic more broadly [48], and considered print and television news media. Earlier articles focused on the 1990 s health care reform efforts (the Clinton Health Security Act) [46, 47] while more recent publications centered around the passage, implementation, and efforts to repeal the Affordable Care Act (ACA) [49, 51, 54, 55, 57]. Studies also revealed frames used to convey information about target populations of health insurance [53] including those on Medicaid [56]. Despite the broad timespan and different policies, articles shared thematic similarities.

One similarity was consideration of the sources included in coverage and the resulting expertise amplified or diminished by media. Politicians were often quoted, as were interest groups such as insurers, while advocacy groups, researchers, or those representing consumers were cited less often [47, 51]. Negative attitudes from business interest groups, such as how universal health insurance coverage would lead to lost jobs (in the case of Clinton health reform) were often referenced (Huebner 1997).

Studies revealed a Limited news media emphasis on the substance of health insurance policy, both in the 1990 s [48] and surrounding the ACA in 2013–2014 [51], focusing instead on political dynamics (such as costs and benefits to stakeholders and politicians). One exception was ACA-related coverage published by African American online newspapers which had more substantive coverage of policy and debate [54]. A prominent example of the politicization of ACA news coverage involves the use of the term Obamacare, which was strategically employed by Republicans as a negative connotation [49] and often used in political-oriented local TV news stories [51]. Some news stories adopted strategies to provide substance and highlight positive effects of the law, such as using specific individuals’ stories of their positive experiences post-ACA passage [57]. Overall, the politicized nature of the ACA debate emphasized partisan associations that perpetuated political heuristics to interpret the law, and these themes persisted into news coverage in 2018 [55].

Other studies identified the frames used by news media in stories about health care costs, health insurance, and Medicaid. One common theme was using values-based framing, such as frames of compassion and cost control used in coverage of health care for undocumented immigrants [53]. Kim et al. (2017) found that placing blame on patients for their rising health care costs cues the audience to view the issue as a personal problem, consistent with personal responsibility values [52]. Value framing was emphasized in other news content about health care, such as the use of individual responsibility framing to shift the burden of high health care costs from system-level factors (e.g., high costs to develop pharmaceuticals) to unhealthy patient lifestyles and the use of health vigilantism, or punishing those who fail to maintain a healthy lifestyle [50]. Other values-based framing included an emphasis on liberty and fairness in stories detailing opposition to Medicaid expansion, while supportive media coverage used care and harm framing [56].

Authors emphasized the medium-specific constraints of reporting on health insurance policy, such as local TV news coverage having difficulty capturing depth or equity implications [55]. This may be attributed to TV news coverage being a visual medium that relies on imagery and simple stories that can be conveyed concisely, challenging the communication of complex issues [46]. Health equity was not a topical focus in these studies. The few journalism implications offered across the studies focused on increasing substantive coverage of the benefits of policy [51] and the use of community-based news reporting to elevate the importance of health insurance policy to the communities they serve [54] .

### Food assistance, hunger, and nutrition

Articles categorized in this topic area were published between 2012 to 2022 and covered the Supplemental Nutrition Assistance Program (SNAP) and school lunches. While the four articles rarely provided specific recommendations for journalists, each emphasized that media coverage of food assistance, hunger, and food security influence the public and policymaking. For example, the language used to describe programs related to food assistance have the potential to cue the need for structural change (e.g., policy as solution) or stigmatization (e.g., programs are rampant with fraud). Authors also identified differences in frames depending on the political ideology espoused by the groups speaking about or producing content. Liberal-leaning messaging is more likely to focus on the broader issues related to hunger and food insecurity, such as poverty [58], and is more likely to use terms such as ‘food security’ instead of ‘hunger’ to describe the nutrition-related consequences [69]. Conservative-leaning messaging is more likely to frame the issue and solutions around the individual through a focus on fraud and work requirements in food assistance programs [58]. As with the welfare studies, studies examining food assistance also noted the potential for negative associations about beneficiaries to create negative associations about the policies, or of the government. News coverage of school lunches commonly focused on the shaming beneficiaries face, rather than contextualizing the widespread problem of childhood food insecurity [70].

This topic area included the single article that focused on COVID-19 [59]. This analysis compared news media from a period just before and during the pandemic and suggests that more coverage published during the pandemic focused on dispelling misconceptions about food assistance programs, broadening public understanding of all who benefit, and discussed solutions and the role of state or federal policy. Language surrounding fraud decreased meaningfully in news media coverage of food assistance programs during COVID-19 and more stories highlighted the difficulty faced by individuals and families “just like us”, as more people used these programs [59]. The shift in coverage during the pandemic suggests there is potential to frame government intervention as a solution through empathetic news coverage of hunger and food insecurity.

### Other social policy/inequality

Articles categorized in this topic area range in publication date from 2002 to 2022 and covered topics related to children’s issues [60], racial and ethnic inequalities [62, 71], and community-level social cohesion [61]. Despite the diverse foci, articles converged around two themes: that different types of news outlets offer different frames to package issues related to inequality, and that contextual information is often lacking. Biswas and Kim [62] noted that ethnic papers, more than mainstream newspapers, were more likely to identify the systemic factors underlying causes of racial unrest and feature community members as sources, while the local newspaper examined relied on official and organizational sourcing. Similarly, Rasmussen [71] noted more in-depth attention to context and systemic factors in the Black press. Kim et al. [61] noted variation in how local news outlets report on issues related to social solidarity (e.g., poverty, immigration) based on the levels of pluralism in those communities.

Each of the articles noted that mainstream media sources, compared to ethnic or other alternative media, limit coverage of contextual information, whether about children’s issues [60] or causal explanations for disparities [71]. Kunkel et al. [60] found that episodic framing is, by far, the primary frame used for stories on youth crime and violence, but that reporting on other children’s issues tends to place the topic in a broader policy environment. As noted in other policy topics, journalists covering racial and ethnic disparities must detail causes to provide necessary contextual information to help readers interpret these issues systemically without resorting to individual stereotypes [71]. However, while mainstream media coverage of racial disparities did describe systemic causal factors, they most often framed these causal attributions as incomplete or pending confirmation, rather than definitive [71].

### Housing/homelessness

The three studies on housing and homelessness were published between 2010 to 2021, tackling issues surrounding the root causes of homelessness, policy responses to homelessness, and framing of structural considerations (e.g., affordable housing and gentrification). All studies analyzed print news content, though they differed in geographic focus: one focused on Northern California [64] while the other two included print news from across the U.S. [63, 65]. Each article raised the concern that the construction of homelessness as an individual problem obscures structural causes of homelessness – for example, news coverage that questions why unhoused people are not in shelters shifts readers’ focus to personal choices, and away from structural issues such as local housing costs, incomes, or shelter availability [63].

From an equity perspective, news coverage that focuses on homelessness at the individual level is more likely to either dismiss racial equity or invoke racist stereotypes. Although race is rarely explicitly mentioned in news stories about housing-related public policy, race and ethnicity are often invoked in coverage of encampments, substance use, sweeps, violence, and health issues [64, 65]. Focus on individual-level causes of homelessness in the media has the added potential consequence of advancing philanthropic or charitable solutions, as opposed to structural solutions that address affordable housing and other root causes [65].

Similar to the other topic areas, framing of homelessness and housing issues in the media also varied based on the actors who shape news narratives. For example, advocacy organizations pushing for social change often sponsor news articles that frame homelessness as a social problem, while articles triggered by research studies focus on “general-level causes and solutions,” and include more statistical evidence [63]. Policymaker sources convey solutions in news coverage related to legislation more so than in stories about the broader housing crisis [64]. Organizations pushing for social change are more likely to present homelessness as a social problem in news coverage, while articles triggered by crimes and conflicts are less likely to do so [63]. Public health practitioners or health researchers are rarely quoted in stories about housing and homelessness, and very few news articles name health as relevant to homelessness, this poses a challenge for researchers seeking to strengthen narratives about housing as a public health issue [63, 64].

News media framing of housing insecurity and homelessness in the U.S. generally fails to sufficiently discuss the intersections of different structural causes. In one analysis, nearly 9 in 10 news stories about housing insecurity focused exclusively on either homelessness, gentrification or affordable housing, leaving the relationships between these issues unaddressed [65]. Publications in this topic area did include recommendations for journalists to prioritize coverage of structural causes – as this framing can lead audiences to consider structural solutions – and steer away from focusing on individual choices and philanthropic solutions that inadvertently distract the public from systems-level solutions that compel governmental intervention [63, 65]. Another recommendation was to strengthen communication between public health practitioners and journalists to advance the connection between housing and health equity [64].

### Paid family leave

Only two articles focused on paid leave. In one, researchers analyzed local television news coverage of paid leave in the United States during 2018 and 2019 [67]. The other article focused on business magazine and print newspaper coverage of a specific case in 2012–2013, Marissa Mayer’s maternity leave while she was the CEO of Yahoo [66].

Tait et al. [67] noted that local TV news stories infrequently provided information that could aid in viewers’ understanding of important policy details related to who would be eligible for benefits and what those benefits would be. The other study focused on questions of how Mayer was portrayed as a mother and company leader, the privilege that she was afforded in accessing maternity leave, and ways the media scrutinized her actions as CEO during that leave [66].

Each article detailed disparities in access to paid leave and the economic and social consequences of this disparity. The article by Tait et al. [67] was one of few articles, overall, to explicitly discuss the health equity-promoting potential of this non-health policy domain. Grandy, in contrast, elevated the implications of the policy for gender equity in the workplace. Neither article included specific recommendations for journalists to consider in covering paid leave policy.

### Early childhood education and care (ECE)

Only a single publication on news coverage of ECE was included. In a 2006 report, a team of researchers analyzed coverage of early childhood education (defined here as pre-kindergarten) from 23 major daily newspapers in the U.S., with the goal of exploring themes of typical coverage, as well as how newsroom standards and practices affect coverage [68]. Recommendations for journalists covering the topic and the health equity implications of content were not a focus. This study noted that new developments in ECE have gone largely uncovered by major newspapers and this is distinct from changes in other fields, such as business and technology. The authors highlight a pattern of coverage that is largely superficial, detailing government actions (e.g., expansions to a state-level pre-k program) instead of content that may build understanding of the potential of ECE, such as a story about research revealing the long-term health impacts of pre-kindergarten. Further, the authors emphasize that coverage diminishes when conflict (e.g., a disagreement over ECE-related legislative spending proposals) ceases, suggesting that stories may be more sensational than substantive. The team behind this work suggested ways that ECE is distinct from other forms of education (K-12) – such as the involvement of faith-based communities in providing ECE – and the confusion and challenge this might introduce for journalists who specialize in the K-12 education context who are looking to cover ECE. Kunkel et al. [60] also commented on the limited attention to ECE relative to news coverage of other children’s issues.

## Discussion

This is the first study to comprehensively examine how the U.S. news media has covered the social safety net, a set of policies and programs that address the social determinants of health, spanning 60 years of news. While the range of social problems and policies encompassed was diverse, there was significant cross-issue consistency that is useful for understanding the political dynamics. This analysis also illuminates gaps in the research literature as well as opportunities to better promote a population health lens in news reporting on these issues.

Considered comprehensively, the body of literature described tensions around journalists’ depictions of these issues in an episodic and individualistic manner versus more systemic attributions of the problems, rooted in history, power, and social structures. While much of the news coverage focused on the individual-level, over time, more news coverage reported on systemic issues, although there was great variability across news outlet type and the sources that contributed those systemic perspectives. Social safety net programs – from housing supports to food programs – are often presented in the news as solutions to individual issues; the structural nature of these issues (e.g., racism, poverty) was less often discussed. Furthermore, other than in news articles about health insurance and/or racial disparities in health, considered on aggregate, neither the news articles themselves, nor the researchers studying these issues, made explicit references to health. This lack of attention to health among studies of domains that are highly relevant to the social determinants of health (such as housing, income supports, and food/hunger) indicates a persistent lack of population health expertise reaching reporters or editors who construct the news. Likewise, across all but a small selection of the sample, we observed a notable absence of health equity frames, both explicit and implicit (i.e., acknowledging systemic causes of racial health disparities). As a consequence, articles about the social safety net may obscure the structural barriers which disproportionately expose marginalized populations to health risks, reinforcing racist and stigmatizing stereotypes and dampening support for equity-oriented policy solutions. Articles lacking broad justifications for intervening and funding the social safety net – such as how it might affect health, longevity, or wellbeing of different marginalized populations – combined with a common focus on individualizing episodes and exemplars – together might contribute to some of the stasis in U.S. policy action on these issues.

This scoping review also uncovered important implications related to the body of scholarship. First, the research is published in a huge range of journals across many disciplines, meaning that scholars studying news media or the social construction of social or health issues may not be familiar with extant work – even if highly relevant – as it may be published in journals with which author teams are not familiar. For instance, a sociologist may be familiar with the journal *Social Problems* but not expect to find a related study in *JAMA*. Different methodological norms (i.e., qualitative/interpretative versus quantitative/computational) also pose a challenge to integrating key findings across so many disparate disciplinary conventions. Second, most existing scholarship concerns health insurance or welfare, not the many other social safety net domains. The literature base would also benefit from more recent analyses of how target populations are described, to assess whether racialized depictions of “undeserving” beneficiaries – a common theme in news media coverage of many of these issues in the 1990 s and earlier – persists. Third, a few major conceptual models remain dominant across studies, such as attention to episodic versus thematic framing (e.g., [16]). While this conceptual lens is no doubt important – and its re-appearance in scholarship across disciplines and across topics is a testament to its significance – with the dramatic changes in news media fragmentation and how audiences consume news media, more conceptual models to interpret news media framing conventions would enrich the literature. Fourth, scholars studying the media must adapt to address news coverage in a wider range of outlets that reach diverse consumers. The majority of studies examined print news media outlets, likely because this is the source that is (still) the most convenient to access and analyze, whether with human-coding or computational methods. That said, news consumers have increasingly moved away from print news [72] toward social media sources or cable news outlets with more polarized content (e.g., Fox, MSNBC), or to podcasts subject to little, if any, editorial oversight [73]. Few studies examined local TV news broadcasts, which remains both a highly trusted news source for health-related information [74], is more uniformly watched across the political spectrum [75], and convey local community concerns. Television broadcasts can also convey implicit information about policy beneficiaries or users of the safety net through images, which print news does not do as readily. Further, social media companies now have major control on how news gets distributed to consumers, driving attention in ways not addressed in this body of literature (see, e.g., [76]), this raises the possibility of even more sensational and/or stigmatizing content about vulnerable populations reaching the public.

This scoping review has Limitations. First, our searches ended in the fall of 2022, so no studies published since then were included. In addition, we found most of the studies identified were older—with a plurality covering the 1990 s and early 2000s. By definition, this means that the studies did not address more recent media phenomena: such as increasing fragmentation of news, selection of news outlets based on partisan predispositions, declining trust in media institutions that was accelerated following the COVID-19 pandemic, the rise in digital news and social media, and use of generative AI technologies for content [77]. Second, while we used a variety of databases (Scopus and Google Scholar) and many keywords to identify a wide net of articles to screen for inclusion, we still may have missed some relevant ones. It was sometimes a subjective judgment about whether a study was sufficiently relevant to the social safety net (such as a study examining coverage of a particular health issue, like diabetes or depression), so while such studies may have added to our understanding of related issues in public health, they were not included. We suggest future researchers continue to analyze news media across issue areas for the insights such reviews provide for policy, politics, research, and journalistic practice.

## Conclusions and implications for journalists

Despite these studies offering rigorous analysis of journalistic practice, to our surprise, studies rarely considered the constraints that news producers face or provided explicit recommendations for journalists themselves. This suggests that journalists are not often considered the audiences of the research, with rare exceptions (e.g., [46, 64]). There is a missed opportunity for this body of work to connect with journalists and/or journalism professional organizations, to make explicit recommendations about how to enhance coverage or reach different types of sources that are informed by journalistic practice. For example, our study suggests that journalists covering social safety net issues – even when these issues are not typically considered health-related [78] – consider population health researchers as key sources in detailing the health impacts of funding cuts or policy decisions that threaten access to housing, paid leave, early childhood education, and programs to reduce poverty. Even within the issue area of health insurance, our analysis suggests that journalists are sourcing politicians or other interest groups more often than sources with the health research expertise to address the health impacts of proposed funding cuts or expansions, beyond the economic or political implications. A concerted effort by journalists and editors to prioritize perhaps nontraditional sources with health expertise over sources with institutional power could enhance coverage of the social safety net, particularly with respect to health equity. Further, these implications are also relevant to journalists outside of the traditional “hard news” beats (and included in this scoping review), such as those described as “soft” news or lifestyle journalism. Lifestyle journalists, whether in traditional news or social media and blogs [79], also convey information about populations affected by the social safety net, and should also be considered as potential avenues for informing public discourse and debate. Given more recent and consequential threats to the social safety net in the U.S. (e.g., One Big Beautiful Bill Act introduced in Congress in 2025), researchers conducting work on the population health implications of any of these social safety net domains should consider more strategic dissemination strategies to reach a diverse set of journalists working across a range of news media outlets and contexts, thus helping to shift agendas and framing conventions within the news.

## Supplementary information

Supplementary Material 1.

## Data Availability

The datasets used and/or analyzed during the current study are available from the corresponding author on reasonable request.

## Abbreviations

SNAP: Supplemental Nutrition Assistance Program
SSDI: Social Security Disability Income
ACA: The Affordable Care Act (more formally, the Patient Protection and Affordable Care Act)
TANF: Temporary Assistance for Needy Families
ECE: Early childhood education
