# Supplementary material for: News media coverage of the U.S. social safety net: themes and gaps from a scoping review
Source: BMC Public Health. 2025 Sep 30;25:3163. doi: 10.1186/s12889-025-23875-x (PMC12487278; doi:10.1186/s12889-025-23875-x)
Supplement: Supplementary file 1 — Supplementary Material 1. [file 12889_2025_23875_MOESM1_ESM.docx]

**Supplementary Appendix (Online)**

**Appendix Table 1 – Keyword Searches**

| **Terms Applied in Scopus and Google Scholar** |
| --- |
| “poverty and media” |
| “safety net” AND “media” |
| “social safety net” AND “media” |
| “welfare” AND “media coverage” |
| “child care” AND “news media” |
| “early care and education” AND “news media” |
| “early childhood education” AND “news media” |
| “food insecurity” AND “news media” |
| “food stamps” AND “news media” |
| “housing” AND “news media” |
| “Medicaid” AND “news media” |
| “paid family leave” AND “news media” |
| “preschool” AND “news media” |
| “safety net” AND “news media” |
| “social policy” AND “news media” |
| “Supplemental Nutrition Assistance Program” AND “news media” |
| “welfare” AND “news media” |

**Appendix Table 2 - Data Abstraction Form**

| **Variable** | **Description** |  |
| --- | --- | --- |
| Name of reviewer | Multiple choice |  |
| Citation | Open text |  |
| Year published | Open text |  |
| Number of authors | Numeric field | Range 1-9+ |
| Disciplinary orientation of authors | Check all that apply | Communication/Journalism; computer science; public health/health sciences; public policy/public affairs; political science/government; sociology; other (describe) |
| Inclusion | Yes / maybe/ no |  |
| Methods of study | Multiple choice | Qualitative only; quantitative only; mixed; unsure/other (describe) |
| Type of content analysis | Check all that apply | Human coding; machine coding; not applicable/other |
| Years of study | Date |  |
| Type of media included | Check all that apply | Print/online; TV news; magazines; social media; entertainment; other |
| Type of media | Open description |  |
| Geography of media | Check all that apply | US/national; comparative (US and another nation); smaller than national (i.e., local news) |
| Geography of media detail | Open description |  |
| Main topic included | Check all that apply | Food assistance/hunger/nutrition; housing/homelessness; health insurance/Medicaid; Early childhood education; paid leave/family leave; poverty/welfare/SSDI; general social policy deservingness; other |
| Main topic included detail | Open description |  |
| Topic: COVID-19 | Yes/no | Reference to whether the research concerns COVID-19 |
| Research questions | Description | From authors’ statement |
| Relevance determination | 5-point scale | Not at all relevant to very relevant to U.S. social policy safety net |
| Key findings 1 - overall | Description | From authors’ text |
| Key findings 2 - implications for equity | Description | From authors’ text |
| Key findings 3 - insights for target / affected populations | Description | From authors’ text |
| Key findings 4 - reference to health or population health | Description | From authors’ text |
| Implications: recommendations for journalism | Description | From authors’ text |
| Implications: recommendations for policy or practice | Description | From authors’ text |
| Implications: recommendations for future research | Description | From authors’ text |
| Overall implications for the project | Description | Analysts’ interpretation |
| Issues/questions/concerns | Description | Flag for further review |

**Appendix Table 3 - Included Articles, Key Categorical Variables (N=40)**

| Main topic | Authors (year) | Title and Journal | Type of research study (qual, quant) | Type of content analysis (human, computer, both) | Years of study | Type of media | Geography of media |
| --- | --- | --- | --- | --- | --- | --- | --- |
| Poverty/welfare/Social Security Disability | | | | | | | |
| 1 | Iyengar, S. (1990) | Framing responsibility for political issues: The case of poverty. *Political Behavior* | Mixed | Human | 1981-1986 | TV news; other not specified | US general/national media |
| 2 | Gilens, M. (1996) | Race and poverty in America: Public misperceptions and the American news media. *Public Opinion Quarterly* | Mixed | Human | 1988-1992 | TV news; magazines | US general/national media |
| 3 | Clawson, R. A.; Trice, R. (2000) | Poverty as we know it. *Public Opinion Quarterly* | Quantitative | Human | 1993-1998 | Magazines | US general/national media |
| 4 | Lawrence, R. (2000) | Game-framing the issues: Tracking the strategy frame in public policy news. *Political Communication* | Mixed | Human | 1996 | Print or online newspapers; magazines | US general/national media |
| 5 | Schram, S. F., & Soss, J. (2001) | Success stories: Welfare reform, policy discourse, and the politics of research. *Annals of the American Academy of Political and Social Science* | Qualitative | Human | 1998-2000 | Print or online newspapers | US national/general media |
| 6 | Yoo, G. J. (2002) | Constructing deservingness: Federal welfare reform, supplemental security income, and elderly immigrants. *Journal of Aging & Social Policy* | Mixed | Human | 1993-98 | Print or online newspapers | US national/general media |
| 7 | Luther, C., Kennedy, D., & Combs-Orme, T. (2005) | Intertwining of poverty, gender, and race: A critical analysis of welfare news coverage from 1993-2000. *Race, Gender & Class* | Mixed | Human | 1993-2000 | TV news | US general/national media |
| 8 | Dyck, JJ and Hussey, LS. (2008) | The end of welfare as we know it? Durable attitudes in a changing information environment. *Public Opinion Quarterly* | Mixed | Human | 1999-2004 | Magazines | US general/national media |
| 9 | Kelly, M. (2010) | Regulating the reproduction and mothering of poor women: The controlling image of the welfare mother in television news coverage of welfare reform. *Journal of Poverty* | Mixed | Human | 1992-2007 | TV news | US general/national media |
| 10 | Brown, H. (2013) | The new racial politics of welfare: Ethno-racial diversity, immigration, and welfare discourse variation. *Social Service Review.* | Mixed | Human | 1993-97 | Print or online newspapers | Less than national/local |
| 11 | Rose, M. and Baumgartner, F.R. (2013) | Framing the poor: Media coverage and US poverty policy, 1960-2008. *Policy Studies Journal.* | Mixed | Both | 1960-2008 | Print or online newspapers | US general/national media |
| 12 | van Doorn, B.W. (2015) | Pre- and post-welfare reform media portrayals of poverty in the United States: The continuing importance of race and ethnicity. *Politics & Policy.* | Quantitative | Human | 1992-2010 | Magazines | US general/national media |
| 13 | El-Burki, I., Porpora, D., & Reynolds, R. (2016) | When race matters: What newspaper opinion pieces say about race and poverty. *International Journal of Communication.* | Mixed | Human | 1994-2010 | Print or online newspapers | US national/general media |
| 14 | Epp, D. A., Jennings, J. T. (2020). | Inequality, media frames, and public support for welfare. *Public Opinion Quarterly.* | Mixed | Human | 1960-2015 | Print or online newspapers | US national/general media |
| Health insurance / Medicaid | | | | | | | |
| 15 | Dorfman, L., Schauffler, H., Wilkerson, J., & Feinson, J. (1996). | Local television news coverage of President Clinton's introduction of the Health Security Act. *JAMA.* | Mixed | Human | 1993 | Print or online newspapers; local tv news; other | Less than national/local (California) |
| 16 | Huebner, J., Fan, D., & Finnegan Jr, J. (1997) | “Death of a thousand cuts": The impact of media coverage on public opinion about Clinton's Health Security Act. *Journal of Health Communication.* | Quantitative | Both | 1993-94 | Print or online newspapers | Less than national/local |
| 17 | Brodie, M., Altman, D., Brady, L., & Heberling, L. (2002) | A study of media coverage of health policy 1997-2000. *Columbia Journalism Review.* | Mixed | Human | 1997-2000 | Print or online newspapers; TV news | US general/national media; less than national/local |
| 18 | Hopper, J. (2015) | Obamacare, the news media, and the politics of 21st-century presidential communication. *International Journal of Communication.* | Mixed | Human | 2011-12 | Print or online newspapers, TV news | US general/national media |
| 19 | Kim, S-H,, Tanner, AH, Foster, CB & Kim, SY.  (2015) | Talking about health care: News framing of who is responsible for rising health  care costs in the United States. *Journal of Health Communication.* | Mixed | Human | 1993-2010 | Print or online newspapers; TV news | US general/national media |
| 20 | Gollust, S. E., Baum, L. M., Niederdeppe, J., Barry, C. L., & Fowler, E. F. (2017) | Local television news coverage of the Affordable Care Act: emphasizing politics over consumer information. *American Journal of Public Health.* | Quantitative | Human | 2013-14 | TV news | Less than national/local |
| 21 | Kim, S., Tanner, A., Kim, S., Foster, C., Oh, S., & Chang, J. (2017) | News focuses on individuals for rising health care costs. *Newspaper Research Journal.* | Quantitative | Human | 1993-2010 | Print or online newspapers; TV news | US general/national media |
| 22 | Viladrich A.  (2019) | "We cannot let them die": Undocumented immigrants and media framing of health deservingness in the United States. *Qualitative Health Research.* | Qualitative | Human | 2009-17 | Print or online newspapers | US general/national |
| 23 | Biswas, M., & Kim, N. (2020) | African American online newspapers’ coverage of policy debate on the Affordable Care Act in 2017. *Newspaper Research Journal.* | Mixed | Human | 2017 | Print or online newspapers | US general/national media; Smaller geography than US general (Atlanta and Philadelphia) |
| 24 | Gollust, S., Fowler, E., & Niederdeppe, J. (2020). | Ten years of messaging about the Affordable Care Act in advertising and news media: Lessons for policy and politics. *Journal of Health Politics, Policy, and Law.* | Quantitative | Human | 2008-19 | TV news | US general/national media |
| 25 | Rozier, M., & Singer, P. (2021) | The good and evil of health policy: Medicaid expansion, Republican governors, and moral intuitions. *AJOB Empirical Bioethics.* | Mixed | Human | 2012-18 | Print or online newspapers | Less than national/local |
| 26 | Jahng, MR. & Littau, J. (2022) | From political to personal: Tracking the use of exemplars in newspaper coverage of the Affordable Care Act. *Journalism Practice.* | Quantitative | Human | 2010-17 | Print or online newspapers | US national/general media |
| Food assistance / hunger / nutrition | | | | | | | |
| 27 | Tolley, N. M., & Ibrahim, J. K. (2012) | What’s in a name? Policy implications of an evolving definition of food security. *Food Studies: An Interdisciplinary Journal.* | Mixed | Human | 1974-2009 | Print or online newspapers | US general/national media |
| 28 | Chrisinger BW, Kinsey EW, Pavlick E, Callison-Burch C. (2020) | SNAP judgments into the digital age: Reporting on food stamps varies significantly with time, publication type, and political leaning. *PLOS One.* | Quantitative | Machine | 1990-2017 | Print or online newspapers | US general/national media |
| 29 | Spruance, L., McConkie, M., Patten, E., & Goates, M. (2021). | A thematic analysis of unpaid school meals in the news media. *Journal of Hunger & Environmental Nutrition.* | Mixed | Human | 2012-17 | Print or online newspapers; TV news; Other, not specified | US general/national media; Less than national/local; Comparative: U.S. and other nation |
| 30 | Mejia, P., Mahmood, H., Perez-Sanz, S., Garcia, K., & Dorfman, L. (2022) | “People Like Us”: News coverage of food assistance during the COVID-19 pandemic. *Health Equity.* | Mixed | Human | 2019-20 | Print or online newspapers | US general / national media |
| Other social policy, social inequality themes | | | | | | | |
| 31 | Kunkel, D., Smith, S., Suding, P., & Biely, E. (2002) | Coverage in context: How thoroughly the news media report five key children's issues. *ERIC – Institute of Education Sciences (Report)* | Mixed | Human | 2001 | Print or online newspapers; TV news | US general/national media |
| 32 | Cabrera Rasmussen, A. (2014) | Causes and solutions: Mainstream and Black  press framing of racial and ethnic health disparities. *Howard Journal of Communications.* | Qualitative | Human | 2000-08 & 2009-11 | Print or online newspapers | US general/national media; Less than national/local |
| 33 | Kim, B., Lowrey, W., Buzzelli, N., & Heath, W. (2021) | News organizations and social cohesion in small, large, and global-local communities. *Mass Communication and Society.* | Quantitative | Human | 2017-2019 | Print or online newspapers | US general/national media; less than national/local |
| 34 | Biswas, M. & Kim, N. Y., (2022) | Racial inequalities in Baltimore City and its reflection in diverse news media coverage after the 2015 unrest around Freddie Gray’s death. *Social Development Issues.* | Qualitative | Human | 2015 | Print or online newspapers | US general/national media; less than national/local |
| Housing | | | | | | | |
| 35 | Best, R. (2010) | Situation or social problem: The influence of events on media coverage of homelessness. *Social Problems.* | Quantitative | Human | 1998-2001 | Print or online newspapers | Less than national/local |
| 36 | Nixon, L., Schaff, K., Mejia, P., Marvel, D., & Dorfman, L. (2019) | Equity and health in housing coverage: A preliminary news analysis from Northern California. *Berkeley Media Studies Group (Report)* | Mixed | Human | 2017-2019 | Print or online newspapers | Less than national/local |
| 37 | Borum Chattoo, C., Young, L., Conrad, D. & Coskuntuncel, A. (2021) | “The rent is too damn high”: News portrayals of housing security and homelessness in the United States. *Mass Communication and Society.* | Mixed | Human | 2018 | Print or online newspapers | US general/national media |
| Paid leave / family leave | | | | | | | |
| 38 | Grandy, K. (2016) | Marissa’s choice: Media coverage of Yahoo CEO Marissa Mayer. *Kadin/Woman 2000 – Journal for Women’s Studies* | Qualitative | Human | 2012-13 | Print or online newspapers; Magazines | US general/national media |
| 39 | Tait,M, Bogucki, C.,Baum, L., Franklin Fowler, E., Niederdeppe, J., and Gollust, SE. (2021) | Paid family leave on local television news in the United States: Setting the agenda for policy reform. *SSM-Population Health.* | Quantitative | Human | 2018-19 | TV news | Less than national/local |
| Early childhood education | | | | | | | |
| 40 | McAdams, K. C., & Henry, T. M. (2006). | An analysis of US newspaper coverage of early childhood education. *Hechinger Institute on Education and the Media (Report)* | Mixed | Human | 2000 & 2003 | Print or online newspapers | US general/national media |

Appendix 4 – PRISMA Checklist

**Preferred Reporting Items for Systematic reviews and Meta-Analyses extension for Scoping Reviews (PRISMA-ScR) Checklist**

**Article:** News Media Coverage of the U.S. Social Safety Net: Themes and Gaps from a Scoping Review

| **SECTION** | **ITEM** | **PRISMA-ScR CHECKLIST ITEM** | **REPORTED ON PAGE #** |
| --- | --- | --- | --- |
| **TITLE** | | | |
| Title | 1 | Identify the report as a scoping review. | 0 |
| **ABSTRACT** | | | |
| Structured summary | 2 | Provide a structured summary that includes (as applicable): background, objectives, eligibility criteria, sources of evidence, charting methods, results, and conclusions that relate to the review questions and objectives. | 1 |
| **INTRODUCTION** | | | |
| Rationale | 3 | Describe the rationale for the review in the context of what is already known. Explain why the review questions/objectives lend themselves to a scoping review approach. | 3-4 |
| Objectives | 4 | Provide an explicit statement of the questions and objectives being addressed with reference to their key elements (e.g., population or participants, concepts, and context) or other relevant key elements used to conceptualize the review questions and/or objectives. | 7 |
| **METHODS** | | | |
| Protocol and registration | 5 | Indicate whether a review protocol exists; state if and where it can be accessed (e.g., a Web address); and if available, provide registration information, including the registration number. | 7 |
| Eligibility criteria | 6 | Specify characteristics of the sources of evidence used as eligibility criteria (e.g., years considered, language, and publication status), and provide a rationale. | 7-8 |
| Information sources* | 7 | Describe all information sources in the search (e.g., databases with dates of coverage and contact with authors to identify additional sources), as well as the date the most recent search was executed. | 7-8 |
| Search | 8 | Present the full electronic search strategy for at least 1 database, including any limits used, such that it could be repeated. | 8-9; Supplemental materials (Appendix Table 1) |
| Selection of sources of evidence† | 9 | State the process for selecting sources of evidence (i.e., screening and eligibility) included in the scoping review. | 8-9 |
| Data charting process‡ | 10 | Describe the methods of charting data from the included sources of evidence (e.g., calibrated forms or forms that have been tested by the team before their use, and whether data charting was done independently or in duplicate) and any processes for obtaining and confirming data from investigators. | 9-10 |
| Data items | 11 | List and define all variables for which data were sought and any assumptions and simplifications made. | 10; Supplemental materials (Appendix Table 2) |
| Critical appraisal of individual sources of evidence§ | 12 | If done, provide a rationale for conducting a critical appraisal of included sources of evidence; describe the methods used and how this information was used in any data synthesis (if appropriate). | N/A |
| Synthesis of results | 13 | Describe the methods of handling and summarizing the data that were charted. | 10 |
| **RESULTS** | | | |
| Selection of sources of evidence | 14 | Give numbers of sources of evidence screened, assessed for eligibility, and included in the review, with reasons for exclusions at each stage, ideally using a flow diagram. | 9 |
| Characteristics of sources of evidence | 15 | For each source of evidence, present characteristics for which data were charted and provide the citations. | 14-16; Supplementary materials (Appendix Table 3) |
| Critical appraisal within sources of evidence | 16 | If done, present data on critical appraisal of included sources of evidence (see item 12). | N/A |
| Results of individual sources of evidence | 17 | For each included source of evidence, present the relevant data that were charted that relate to the review questions and objectives. | 14-16 |
| Synthesis of results | 18 | Summarize and/or present the charting results as they relate to the review questions and objectives. | 13-26 |
| **DISCUSSION** | | | |
| Summary of evidence | 19 | Summarize the main results (including an overview of concepts, themes, and types of evidence available), link to the review questions and objectives, and consider the relevance to key groups. | 26-29 |
| Limitations | 20 | Discuss the limitations of the scoping review process. | 29 |
| Conclusions | 21 | Provide a general interpretation of the results with respect to the review questions and objectives, as well as potential implications and/or next steps. | 30-31 |
| **FUNDING** | | | |
| Funding | 22 | Describe sources of funding for the included sources of evidence, as well as sources of funding for the scoping review. Describe the role of the funders of the scoping review. | 32 |

JBI = Joanna Briggs Institute; PRISMA-ScR = Preferred Reporting Items for Systematic reviews and Meta-Analyses extension for Scoping Reviews.

* Where *sources of evidence* (see second footnote) are compiled from, such as bibliographic databases, social media platforms, and Web sites.

† A more inclusive/heterogeneous term used to account for the different types of evidence or data sources (e.g., quantitative and/or qualitative research, expert opinion, and policy documents) that may be eligible in a scoping review as opposed to only studies. This is not to be confused with *information sources* (see first footnote).

‡ The frameworks by Arksey and O’Malley (6) and Levac and colleagues (7) and the JBI guidance (4, 5) refer to the process of data extraction in a scoping review as data charting*.*

§ The process of systematically examining research evidence to assess its validity, results, and relevance before using it to inform a decision. This term is used for items 12 and 19 instead of "risk of bias" (which is more applicable to systematic reviews of interventions) to include and acknowledge the various sources of evidence that may be used in a scoping review (e.g., quantitative and/or qualitative research, expert opinion, and policy document).
